# Exploring why patients in heroin-assisted treatment are getting incarcerated—a qualitative study

**DOI:** 10.1186/s12888-022-03814-5

**Published:** 2022-03-07

**Authors:** Maximilian Meyer, Bernd Rist, Johannes Strasser, Undine E. Lang, Marc Vogel, Kenneth M. Dürsteler, Marc Walter

**Affiliations:** 1 Psychiatric University Clinics Basel, University of Basel, Wilhelm Klein-Strasse 27, 4002 Basel, Switzerland; 2 Faculty of Medicine, University of Basel, Klingelbergstrasse 61, 4056 Basel, Switzerland; 3 Department for Psychiatry, Psychotherapy and Psychosomatics, Psychiatric Hospital, University of Zurich, Lenggstrasse 3, 8008 Zurich, Switzerland; 4 Psychiatric and Psychotherapeutic Clinic, Psychiatric Services Aargau, Königsfelderstrasse 1, 5210 Windisch, Switzerland

**Keywords:** Opioid use disorder, Criminal offense, Crime, Imprisonment, Delinquency, Opioid agonist treatment, Qualitative study, Interview

## Abstract

**Background:**

Heroin-assisted treatment has proven effective in reducing criminal offenses in opioid dependent individuals. Few studies attempted to explain the observed crime reduction and the reasons why these patients keep offending and getting incarcerated have to date not been explored.

**Methods:**

Patients with a history of incarcerations during the time of participating in heroin-assisted treatment (*n* = 22) were invited to a semi-structured, narrative interview. Findings were evaluated with Mayring’s qualitative content analysis framework. Additionally, the Montreal Cognitive Assessment test and the multiple-choice vocabulary intelligence test used to assess cognitive impairment and premorbid intelligence levels.

**Results:**

Three main categories emerged in patients’ narratives on their incarcerations: cocaine use, impaired functioning, and financial constraints. Lifetime prevalence of cocaine use disorder was 95.5% and their cocaine use often led to patients getting incarcerated. Impaired functioning mainly constituted the inability to receive and open mail. Financial constraints led to incarcerations in lieu of payment in 16 participants (72.7%). Categories overlapped notably and often occurred in close temporal proximity. A fourth category on the likelihood of getting incarcerated again in the future was inhomogeneous and ranged from the strong conviction to complete rejection of the scenario. Average premorbid intelligence levels were found, whereas the cognitive assessment suggested severe cognitive impairment in our sample.

**Conclusion:**

Participants mainly reported to have committed minor offenses and not being able to pay for resulting fines. The resulting prison sentences are an unconvincing practice from a medical and economic perspective alike. Public expenditure and the interruptions of the continuum of care could be reduced by legislatively protecting these marginalised patients.

## Background

Heroin-assisted treatment (HAT) was first introduced in Switzerland in 1994 as an alternative treatment option for individuals with severe opioid dependence. It has since been offered in specialised outpatient centres and comprises the supervised administration of medically prescribed diacetylmorphine .(“heroin”, DAM) [1] In Switzerland, access to HAT is limited by specified criteria that patients must meet: in addition to the presence of opioid-dependence for at least two years and the minimum age being 18 years old, patients also need to have had at least two unsuccessful traditional treatment attempts (e.g. methadone-maintenance treatment, MMT) [2].

Since its introduction, HAT has been evaluated in randomised controlled trials in the Netherlands, Belgium, Spain, Germany, the United Kingdom and Canada [3–8]. It has shown to be effective in reducing illegal heroin use and in improving patients’ health status, living conditions and labour market integration [9, 10]. Importantly, treatment retention was observed to be superior in HAT when compared to other forms of opioid agonist treatment (OAT) like MMT [3]. It was also found to be cost-effective, although the intervention itself is significantly more expensive than traditional OAT [11, 12].

Another effect observed in the heroin trials was the reduction of criminal offenses, both in the methadone and the heroin groups, with the HAT group showing significantly superior results in some studies [13]. The German heroin trial observed a greater decline in property offenses and drug-related crime specifically, whereas no between-group differences were found in the reduction of violent crime and fraud [14]. The underlying mechanisms have been explained by the reduction of illegal drug use, less victimisation, and less time spent in the drug scene, which resulted in a reduction of drug-related acquisitive crime. Interestingly, no link between crime reduction and social integration, economic situation, and housing situation was found in the Swiss and German heroin trials [14, 15].

Cognitive impairment could be another factor contributing to the occurrence of incarcerations in this population. Studies have demonstrated a broad range of cognitive impairments in patients receiving MMT [16, 17]. Even though no impairment in long-term memory was observed [18], these findings suggest a marked impact on daily functioning of patients with opioid use disorder. Equally, a higher prevalence of cognitive impairment has been observed in prison populations, when compared to community samples [19] and it has been suggested that cognitive impairment contributes to recidivism and repeated arrests [20, 21]. However, there is a lack of studies reporting predictors of incarcerations in patients receiving OAT. Research has to date not yet explored the possible link between incarcerations and cognitive impairment in this population, even though an estimated 36% of people who inject drugs also have a history of incarceration in Western Europe [22].

Previous literature concluded that by eliminating the need to acquire illicit heroin a reduction in criminal acquisitive behaviour is observed as well. However, it remains unclear as to why patients in HAT keep offending and are consequently getting incarcerated. This is highly relevant from both an economic and a patient perspective. First, incarcerations interrupt the continuum of care, since prisons usually do not offer HAT, leading to patients receiving less effective OAT [23]. Second, the action-taking of the judicial system is expensive and accounts for large sums of public expenditure [12]. Therefore, it would serve the individual patient’s as well as the public’s interest to explore the reasons behind these incarcerations.

Literature reporting on the prevalence of criminal offenses in HAT has to date not specifically explored the underlying mechanisms that lead to criminal offending in patients receiving HAT. This is surprising, since a better understanding of these mechanisms is paramount to reduce crime and prevent offending behaviour in these patients in the future. We therefore aimed to characterise offending patients in HAT and to explore their perspectives on the reason as to why they were incarcerated. We hope to provide insights on how to reduce criminal offenses in these patients and minimise the resulting economic burden. This could be achieved with the expansion of HAT guidelines by developing and integrating preventive strategies or by informing future legislative changes.

## Methods

### Aim

This study aimed to investigate the perceptions of patients in HAT on the reasons why they were incarcerated in the past. Additionally, we aimed to provide further insights on sociodemographic, medical, and neurocognitive characteristics of patients receiving HAT with a history of incarcerations.

### Study Design

A qualitative study was conducted. A semi-structured interview guide was developed by the authors to inquire about the areas of interest for the study aim. Additionally, instruments for the assessment of cognitive impairment and premorbid intelligence were used.

## Setting and Participants

The study was conducted in an outpatient centre in Basel, Switzerland, that specialises in HAT for individuals with severe opioid use disorder. Patients visit up to twice daily for supervised administration of oral, nasal, or injectable DAM. Additional take-home medication comprises slow-release oral morphine and methadone. In case of incarceration of a patient, the medical prescription of opioids through our centre is mandatory for continued OAT in prison. Incarcerations are therefore well documented in the patient information system. Every patient with one or more served prison sentence during participation in HAT were invited to participate.

### Data collection and Analysis

Interviews began with a narrative opening question. The interview guide is provided in Table 1 and was held short on purpose to allow the patients to provide and expand on their narrative. Some statements were followed by nonsuggestive, open-ended questions if it seemed necessary for the course of the interview or clarification. Recurring themes that emerged in early interviews were explored in the following ones, in accordance with qualitative research standards. This ensured maximum variety of emerging categories while also being able to further examine earlier findings.

**Table 1:** Interview guide

Opening question
This interview is about your previous experiences with the penal system, with prisons to be precise. I now ask you to recall the time that led to you having to serve a prison sentence. (Pause) In the next minutes, can you tell me about this time in your life, giving as much detail as possible about how it came about that you had to go to prison?
Follow-up questions (ask if no narrative emerges)
Why did you have to serve a prison sentence?
What would have had to be different in your life to avoid a prison sentence?
Do you think it is likely that you will have to go to prison again in your life? (Why? Why not?)

All interviews were held face-to-face in the months of August and September 2021 and were conducted by MM and BR in an office room at the research site. Both researchers had received training as psychiatrists and had experience in the treatment of substance use disorders. Interviews were held in German and transcribed verbatim by MM. Some participants chose to answer in Swiss German, which is a dialect for which there is no official written standard. These interviews were transcribed verbatim into standard German. Transcripts were assigned a code-letter and potentially identifying information was removed. Interviews lasted between 4 and 27 min with an average duration of 10 min. The length of the interviews was participant dependent. Voice Memos for iOS 14 was used for recording.

Coding and content analysis was carried out in German. The corpus was analysed in accordance with Mayring’s qualitative content analysis [24]. This framework provides a systematic and inductive approach of qualitative text analysis. The data was analysed using an inductive procedure and was approached without presumptions. MM and BR defined selection criteria for codes and the abstraction level of categories. The interviews were then coded separately. BR applied the codes, and all transcripts were blindly coded again by MM to ensure consistency. Codes and emerging categories were discussed and revised by MM, BR, and MW in regular meetings to reach consensus in the analysis. MAXQDA 2020 (VERBI Software GmbH, Berlin, Germany) was used for coding. Quotes that were chosen to be included in the article were translated verbatim into English by MM.

### Additional instruments

To further characterise our sample, additional validated instruments were used.

The Montreal Cognitive Assessment (MoCA) test [25] is a brief paper–pencil tool that assesses seven cognitive domains: visuospatial/executive functioning, language, memory, attention and concentration, calculation, conceptual thinking, and orientation. The maximum score is 30 and a score below 26 is considered abnormal, suggesting cognitive impairment.

The multiple-choice vocabulary intelligence test (Mehrfachwahl-Wortschatz-Intelligenztest, MWT) is a measure of crystalline intelligence and is widely used in the German-speaking part of Europe [26]. It correlates well with global IQ in healthy individuals and aims to assess premorbid intelligence. It consists of 37 items. Each item contains a German word and four similar sequences of letters without meaning. The test was provided in paper–pencil form.

## Results

One-hundred and sixty-five patients received HAT at the research site at the time of study conduction. Twenty-six patients that had been incarcerated during treatment were identified. These patients were invited to the study and 22 agreed to participate. Sociodemographic characteristics, treatment variables, and psychiatric disorders are provided in Table 2.

**Table 2:** Sample characteristics (*n* = 22)

		*n*	%	M	SD
Sociodemographic characteristics					
Sex	female	3	13.6		
	male	19	86.4		
Age				45.14	6.94
Nationality	Switzerland	17	77.3		
	Other	5	22.7		
Living situation	rented apartment	12	54.5		
	care home	8	36.4		
	assisted living	2	9.1		
Source of income	social welfare	12	54.5		
	invalidity insurance^a^	10	45.5		
	employment	-	-		
Relationship status	single	11	50.0		
	in a relationship	10	45.5		
	divorced	1	4.5		
Treatment variables					
Length of treatment in the centre	< 1 year	-	-		
	1–5 years	9	40.9		
	6–10 years	5	22.7		
	11–15 years	4	18.2		
	16–20 years	2	9.1		
	> 20 years	2	9.1		
Psychiatric medication at the time of study conduction	diacetylmorphine	21	95.5		
	morphine-sulfate	19	86.4		
	methadone	2	9.1		
	levomethadone	1	4.5		
	benzodiazepines	13	59.1		
	stimulants	4	18.2		
Route of DAM administration	intravenous	10	45.5		
	oral	6	27.3		
	oral and intravenous	5	22.7		
	not applicable^b^	1	4.5		
Psychiatric disorders (lifetime prevalence as documented in the patient file)					
	Opioid Use Disorder	22	100		
	Cocaine Use Disorder	21	95.5		
	Alcohol Use Disorder	8	36.4		
	Sedative, Hypnotic and Anxiolytic Use Disorder	7	31.8		
	Personality Disorders	5	22.7		
	Cannabis Use Disorder	4	18.2		
	ADHD	4	18.2		
	Major Depression	4	18.2		
	Schizophrenia	3	13.6		
	Anxiety Disorders	2	9.1		
	Disruptive Mood Dysregulation Disorder	1	4.5		
	Bipolar Disorder	1	4.5		

^a^ Patients receiving insurance income due to psychiatric disability

^b^ One patient had stopped their DAM prescription at the time of study conduction but had received DAM during the time of their incarceration;

### Cognitive assessment

All patients (*n* = 22) participated in the MoCA and the MWT assessment. MoCA scores ranged from 6 to 26 points (M = 20.41; SD = 5.92), suggesting a very high prevalence of cognitive impairment in our sample. MWT scores ranged from 20 to 34 points (M = 28.45; SD = 3.31), suggesting average premorbid intelligence levels.

### Interview analysis

**Fig. 1:**
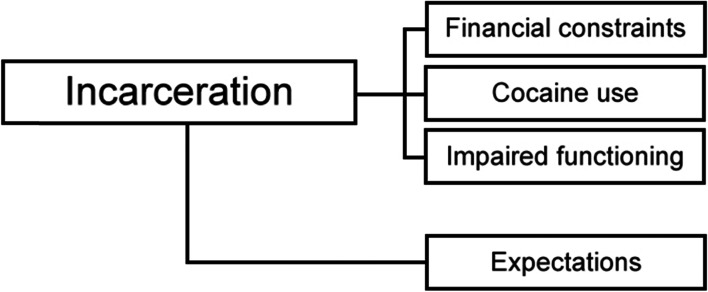
Main categories

The number of documented incarcerations during HAT varied from one to three and multiple patients reported that they had been incarcerated previously to HAT initiation as well. In these cases we specifically asked patients to recall prison sentences served at the time they had already received HAT. We identified three main categories in patients’ narratives: financial constraints, cocaine use, and impaired functioning. These categories represent factors that – from the patients’ perspective – contributed to their past incarcerations. There was a notable overlap among these categories, which is followingly also described in detail. A fourth category (expectations) emerged in the analysis of the follow-up question that asked whether patients deemed it likely to serve a prison sentence in the future. Figure 1 provides an overview of the categories.

### Financial constraints

Most frequently, patients reported to have been fined without being able to fulfil the resulting request for payment. The most common reason for fines was fare evasion in public transport. Out of 22 participants, a total of 16 (72.7%) reported to have been incarcerated due to fare evasion. Other law violations participants were fined for comprised unauthorised waste depositing, and bike-driving at night without lights.

Yes, it was a tramway fine and back then I was still really on it, on drugs and everything and drove without paying a lot- actually always without paying. And if you are unable to pay then it goes further and further and then that stacks up, and this stacks up, and then sometimes for two fines- there are 1000 francs coming. (C, female)

Participants reported to have had the option of paying their fines at the time of their arrest. However, not all chose to do so because they were against paying in principle. Others stated that they would have bought themselves out but were not able because they did not have enough money at the time.

Yes, that was last year. I was in [prison] then, also because of a tramway fine. I would have been sentenced to 25 days, 30 francs [day rate]. But I was able to pay myself out with all the money for the month from social services, after five days. (S, male)

That was tramway fine of 150 francs. Converted to one day of prison. Can you imagine? Converted in one day of prison, right? And then the commissioner came by and asked whether I could pay or not. Then I said: ‘No!’. Although, I could have, but oh well, I thought: ‘No!’. (D, male)

Another reason that emerged to contribute to fare evasion was homelessness, with one patient reporting to use public transport to sleep in.

But the bad thing is that when you don’t have anything to sleep in and it gets cold, you must go in somewhere. Then you have almost no choice but to get into a tramway. There were friends who got on trains, but that's more stupid, because suddenly you get up and you’re in Milan. (K, male)

Financial problems led patients to evaluate their priorities on how they want to spend their money, deeming consumer goods more important than the risk of getting caught.

Yeah, [I had] no money in the bag. Just before payday. Most of the time, when you have 10 francs left in your bag, you think, ‘Yes, I still have to have cigarettes and tobacco. Maybe I’d also like to have a beer, or something. Yes, come on, it [driving without paying] could work, couldn’t it?’ (U, male)

Participants repeatedly stated that their fines were cumulated by the prosecution to prolongate the resulting prison sentence. The cumulation of fines also resulted in higher orders for payment which some participants were unable to settle.

Fines used to be tramway fines or, in the very early days, train fines when you went to Zurich and came back. You had to serve all that in prison. The smallest shitty fines. And [public transport] fines. If you drove without paying for a year, you got caught 10 times. That’s about 1000 francs, and if you’re unlucky, you’ll have a 50 francs day rate. Then you’re in jail for 20 or 30 days for nothing. But it also goes up to 100 days within a very short time. (P, male)

Yes, because I was caught two or three times [driving without paying] and they added it up and then- yes. That’s how it came about that I had to go to prison a few years later instead of- Yes, I couldn’t pay that, that’s still- It was a high amount, and I couldn’t pay that. (E, male)

The police usually wait until they - not only until they have one offense, then they leave you alone. But when they have ten offenses together, that is, ten or fifteen offenses, then there is a court hearing, a conviction and then there is a prison sentence, yes. (H, male)

### Cocaine use

Many participants reported to have been incarcerated in connection with their cocaine use. Acquisitive crime included fraud, shoplifting, online purchases on account without paying, prostitution, and drug dealing. Some participants were fined for possession. One participant stated that he had previously already dealt with substances, but his cocaine dependency led to them to conduct acquisitive crime. He also stated that all his legal problems and following incarcerations had begun with his cocaine use.

As soon as I started using cocaine, I became a criminal. Well, I was also dealing before that. (R, male)

Another participant was crossing the state border from Germany to Switzerland but had forgotten that he was carrying cocaine. He was stopped by border control for a routine check and followingly sentenced abroad.

I didn’t even think that I still had this in my pocket. I thought I had already used it all. But then I still had a bit of cocaine in my trouser pocket. (T, male)

Fraud was committed by another participant to finance his cocaine use. He forged cheques to withdraw larger sums of money even though he anticipated that he would eventually get caught.

If you just put a zero at the end or something. It’s quite clear that it comes out, but you put up with it, yes. (P, male)

One participant engaged in prostitution outside of the cantonal tolerance zones (i.e., declared public spaces in which prostitution is not prosecuted by law) and shoplifting to acquire money for their cocaine use.

I was prostituting myself. And that’s when the police caught me. That is not allowed. I mean illegal. That was at [public square] and there they- I got a report because of that. And the second time was because they caught me shoplifting. […] [I did this] for the cocaine as well. Someone had told me that I would get money if I brought him this and that. (M, female)

### Impaired functioning

Cognitive impairment and psychiatric disability emerged as a third category. In hindsight, some participants stated to know that their prison sentences could have been avoided by paying off their fines or doing community service. However, at the time leading to incarceration, they were unable to make these arrangements. Some stated to have felt overwhelmed by their mail and did therefore not open it. Others stated that they did not receive mail with payment reminders at all due to homelessness.

Yes, what should have been different? I should have opened my mail, yes, and looked at it. And I should have considered the possibility of- well, you can work it off, can you not? You can go to work. But if you don’t open the letters, then you can’t. Yes, then it just doesn't work anymore, does it? Then they [police] just stand in front of the door and then it’s- well, then they are not open for discussion anymore. (E, male)

If you always pick up mail and everything, then you don’t go [to prison] - then you can react to these orders. But if you don’t get mail because you live on the street or with a friend, or you don’t have accommodation, then you can’t react. [...] Drug addicts with mail- is not always so good. Yes, they often neglect it, don’t they? Or don’t react. Don’t write back. Yes. (L, male)

I just let it slide. I didn’t think much about paying them at that moment. And I didn’t think about the consequences. So, I was actually really naïve then- well, c'est la vie. Then I’ll just have to do time again. Like that. (T, male)

Because I’ve always received reminders. And another reminder. The last reminder. [...] And somehow, I didn’t have the energy to sort it all out and tackle it. Actually, I would have needed help from someone. (O, male)

Other participants stated that they lost their mail or were unable to open it. This behaviour relieved the pressure they were under but did not provide them with a long-term solution.

Then once the mail- it was gone. The pressure is gone. All the pressure of life is gone at once. Yes, briefly, but it always comes back. You can’t stop it. (V, male)

And I didn’t take care of my mail then. If I ever opened my letterbox, then I put it on the table at home. (U, male)

### Expectations

When asked, whether patients deemed it likely they would serve a prison sentence again in the future, patients´ responses ranged from ruling the possibility out entirely to the strong conviction of getting incarcerated again. Patients deeming it unlikely pointed out that their participation in HAT and support provided by their legal guardian prevented them from further incarcerations. Patients who were convinced to serve a prison sentence again in the future stated that their behaviour, financial situation, and cocaine use had not changed. These patients also stated that prison sentences had no punishing effect on them. Some patients stated uncertainty, reflecting the main category of impaired functioning as they suspected that previous fines might re-emerge without knowing it for sure.

I don't think so, no. Because unlike in the past, it is now a matter of course for me that a bill is also paid. (J, male)

As things stand, I don’t know. Because at the moment I’ve lost the overview of my debts, my fines, a bit - I honestly can’t tell you. But as soon as I get an overview, I will do everything possible to avoid that. (Q, male)

Oh nothing, I don't really care if I go to prison or not. I'm going to prostitute myself anyway. (M, female)

### Interconnecting the categories

During the analysis we found a notable overlap in the identified categories. The category of impaired functioning was strongly interconnected with cocaine use and financial constraints. This interconnection presented itself by a close temporal proximity of the occurrence of more than one category in some patients, whereas in other participants one category seemed to condition the other.

The following quote provides an example of the proximity of all three categories in one patient. She described using cocaine, her impairment of functioning, and financial constraints. This was followed by fare evasion which then led to her incarceration.

Yes, if you take drugs [cocaine] and so on and quickly, quickly and hastily – that’s why I don’t have that anymore, that quickly, quickly, quickly, right. And if you don’t use anymore, then you gain weight. But I prefer not to use anything anymore. I just had to serve time, but I- Yes, everything else is- do you understand- is more important than buying a tramway ticket. (C, female)

Fines for ignoring payment reminders often conditioned impaired functioning. Due to patients being unable to open their mail, they were also unable to respond to the reminders or seek help. Therefore, they had to serve a prison sentence in lieu of payment.

Yes, a fine came and I didn’t pay the fine and then a reminder came, and I didn’t pay the reminder, and then that was converted into another fine. (B, male)

Fines for fare evasion also conditioned impaired functioning. This is exemplified by the following quote of a participant who possessed a valid ticket but did not show it because he forgot to do so.

Most of the time I had a ticket. So, I have my legal guardian. He pays for the annual subscription. And sometimes I don’t have it on me. And sometimes I forget to show it and so- and I get fines. It’s actually my own fault, isn’t it? At some point you forget- actually I should have shown it, so-. (H, male)

There were also instances in which fines conditioned cocaine use. Lifestyle factors associated with substance use and the subjective effects of cocaine led to the neglection of responsibilities and resulted in fare evasion fines.

Because instead of paying for a subscription, I spent the money on drugs [cocaine]. Yes. That’s that. And I didn’t give a shit when I was on it. You don’t give a shit when you’re on it. (R, male)

## Discussion

This qualitative study investigated patients’ perceptions on why they had to serve past prison sentences during HAT participation. We found that patients in our study did not commit heavy crimes such as violent crimes but rather committed fare evasion, drug-related crimes, and property offenses. This is in line with previous literature on delinquency in HAT [13, 14]. Our findings strongly indicate that incarceration in patients receiving HAT is associated with impaired functioning. On the one hand, this can be attributed to cocaine use. On the other hand, patients in HAT often suffer from severe chronic somatic and psychiatric comorbidities [27]. Impaired functioning in this population is also reflected in their employment status and living situation, as all of them depended either on social welfare or received invalidity insurance at the time of study conduction (see Table 2). The results of our cognitive assessment further support this observation, as the average MoCA score of our sample was 20 points, whereas a score of 26 or higher is considered normal. However, we found the MWT scores in our sample to be average. MWT scores correlate well with global IQ in healthy individuals and aim to measure premorbid intelligence through knowledge assessment [26]. Average premorbid intelligence levels implicate that the impairment of this population resulted from substance use-related lifestyle factors, which are likely to play a larger role in the occurrence of incarcerations than pre-existing conditions and social upbringing.

Remarkably, 21 of 22 participants (95.5%) suffered from cocaine use disorder. Unlike with opioid use disorder, no pharmaceutical treatment for cocaine use disorder is approved to date [28], making treatment particularly challenging. During our analysis, we found a noticeable overlap of cocaine use with the other categories and some patients reported these factors to have occurred together during the time leading to their incarceration. This underlines the association of incarceration with the interplay of substance use, financial constraints, and other related socio-economic and medical factors like societal marginalisation, co-occurring psychiatric disorders, and homelessness. The substance use-related lifestyle is associated with impaired functioning, impulsivity, and high levels of risk-taking [29]. These disadvantageous traits negatively influence decision-making processes which has been explained by the devaluation of long-term rewards in favour of short-term gratification [30]. The combined occurrence of financial constraints, substance use, and impaired functioning also favours the arising of situations in which action-taking is no longer possible. In these cases medical, social, and psychological support systems, like those provided in OAT centres and harm reduction facilities are necessary to break the downward spiral and avert further harm.

Women with substance use disorders are particularly vulnerable and the link between prostitution and substance use has been described in literature early on [31]. With regards to gender differences observed in our sample, we found limited evidence for differences in the contributing factors that led to their incarcerations. However, even though only three women participated in our study, one reported to have engaged in sex work which then led to her having to serve a prison sentence. We therefore suspect that the observation of voluntary and coerced sex work would have become more apparent in a larger sample of patients from multiple HAT centres.

Patients’ views on whether they deemed it likely to serve a prison sentence again in the future differed vastly. Some had sought help and recognised the importance of support in legal matters, which led them to rule out the possibility entirely. Other patients stated that they did not really care about serving prison sentences at all, as they did not have any punishing effect on them. This suggests that incarcerating these patients cannot achieve a change of behaviour. Making them serve prison sentences for minor offenses like fare evasion therefore appears an unconvincing practice.

In Switzerland, there are many OAT centres and treatment slots available when compared to other developed countries [32], and patients are usually able to return to their previous treatment regimen following release. Nevertheless, incarcerations of these patients interrupt the continuum of care, as prisons usually do not offer HAT [23]. Patients reported to have served multiple short prison sentences, which had destabilised their everyday life, daily routines, and led to delayed and inferior medical treatment. However, the practice of incarcerating opioid dependent individuals for minor offenses is problematic from a medical perspective. In other western countries, research has demonstrated that prison sentences predict the engagement in high-risk substance use behaviours [33] and consecutive disease transmission [34]. This risk is further elevated as in some countries, the judicial prosecution of illicit substance use facilitates incarcerations of these highly vulnerable patients. Therefore, prison sentences for minor offenses are also unconvincing from an international, public health perspective.

From the economic point of view, incarcerations in lieu of payment are even more doubtful in this patient population. Data from the Federal Statistical Office shows that in 2019 more than half of all prison sentences in Switzerland were served in lieu of payment [35]. In 2013 the Swiss Federal Council published a report, estimating the cost of one prison day per inmate at 390 Swiss francs [36]. Saving these large sums of public expenditure is very likely much more cost-effective when compared to the current practice.

Thus, the question arises whether the current way of dealing with patients in HAT who are unable to pay fines can be improved. The severity of the psychiatric impairment in this population is exemplified by one patient, who reported to have been fined for fare evasion, even though he possessed a valid ticket. He was overwhelmed with the situation and therefore unable to show it to the controlling ticket officer. Furthermore, every patient in our sample was unemployed and depended on social welfare or received invalidity insurance. Also, data from the Federal Statistical Office and the Swiss Federal Council provide an estimate of the size of public expenditure on the penal system. These data are likely underestimating the total expenditure as additional cost generated by other institutions of the judicial system (e.g., police costs and administration of fines) are not considered. Thus, other solutions are likely more cost-effective. One such solution could be the subsidising of public transport tickets for these patients. Another solution could be waiving of fines for this population if they are unable to pay, especially if they have already previously been incarcerated in lieu of payment. In these cases, prison sentences have not led to a change in behaviour but are costly and interrupt the continuum of care.

### Limitations

As this was a qualitative study, we examined patients’ views and perspectives on the reasons of why they were incarcerated. We did not obtain police records or any judicial documentation to verify the respective reasons for incarcerations or the exact number of (lifetime) incarcerations. However, due to prison sentences requiring the prescription of opioids for continued OAT, we mostly observed comparably short prison sentences, compatible with incarcerations in lieu of payment. The MWTs suitability in assessing premorbid intelligence in conditions such as dementia has been debated [37], whereas no data on its validity exists in opioid use disorder with heterogenous comorbidities. Also, our analysis presents logical limitations as the categories of “impaired functioning”, “financial restraints”, and “cocaine use” are unequal in their level of abstraction. Nonetheless, we decided to retain this analytical construct, as in our opinion it describes the distinct phenomena comprehensibly. Finally, our interview guide was held short, leading to brief narrations of participants when compared to other qualitative studies. More extensive in-depth interviews would likely have resulted in a richer corpus and allowed for a more meaningful analysis.

## Conclusions

Patients in HAT reported to be mostly incarcerated for minor offenses like fare evasion. Impaired functioning, financial constraints, and cocaine use either occurred in isolation or together in close temporal proximity and contributed to the following incarcerations. Support systems like participation in HAT, help provided by social workers, and legal guardianship were found to reduce the risk of repeated incarceration from patients’ perspectives. Future quantitative research is needed to identify predictors of incarceration in OAT and HAT-populations. An observational multi-centre study could include additional sources of data (e.g., police records) while also controlling for factors like psychiatric comorbidity, age, and gender. We conclude that prison sentences for fare evasion in these highly marginalised and severely impaired patients are not useful from an economic and medical point of view, as they are not cost effective and interrupt the continuum of care.

## Data Availability

The datasets generated and analysed during the current study are not publicly available due privacy concern but are available from the corresponding author on reasonable request.

## Abbreviations

HAT: Heroin-assisted treatment
DAM: Diacetylmorphine
MMT: Methadone maintenance treatment
MoCA: Montreal Cognitive Assessment test
MWT: Multiple-choice vocabulary intelligence test
